# New Appliances and Things Medical

**Published:** 1895-09-07

**Authors:** 


### NEW APPLIANCES AND THINGS MEDICAL.

[We shall be glad to receive, at our Office, 428, Strand, London, W.C., from the manufacturers, specimens of all new preparations and appliances which may be brought out from time to time.]

### BIPALATINOIDS.

(Messrs. Oppenheimer.)

The latest development in these most ingenious double capsules is the manufacture of them containing the materials for urine-testing. Bipalatinoids of Fehling's, Pavy's, and the ferrocyanic tests have all been carefully tested by us. They give the required reactions with ease, and as each one of the sugar tests is put up of a definite strength a very fair estimation of the percentage composition can be obtained. As the covering differs from that of the medicinal palatinoids, and is of a neutral composition, it has no effect in the reaction desired. In order to use the sugar tests, all one has to do is to drop one into a little of the suspected fluid and boil, when the usual reactions occur if sugar be present. The ferrocyanic albumen test requires to be cut across before using. The ease with which these tests can be used, their small bulk, portability, and permanence certainly warrant us in saying that Messrs. Oppenheimer have made yet another advance in the armamentarium of the physician. Besides these forms of palatinoids, the firm have recently introduced those containing thyroid powder equivalent to five grains of the fresh gland, also palatinoids of other animal substances whose keeping properties are uncertain. This again is a distinct advance, as owing to the impermeability of the envelope, the easily putrescible powder is kept as fresh and strong as when first put up.

